# Drugs foresight 2020: a Delphi expert panel study

**DOI:** 10.1186/1747-597X-9-18

**Published:** 2014-05-03

**Authors:** Tomi Lintonen, Anne Konu, Sanna Rönkä, Elina Kotovirta

**Affiliations:** 1 Finnish Foundation for Alcohol Studies, PO Box 30, FI-00271 Helsinki, Finland; 2 Tampere School of Health Sciences, FI-33014 University of Tampere, Tampere, Finland; 3 Department of Social Research, University of Helsinki, PO Box 16, FI-00014 Helsinki, Finland; 4 Ministry of Social Affairs and Health, PO Box 33, FI-00023 Government Helsinki, Finland

**Keywords:** Drug policy, Foresight, Expert panel

## Abstract

**Background:**

Historically substance misuse has been relatively common in western countries, but comparatively few Finns report drug use. The Drugs 2020 study aimed at foreseeing changes in the drug situation in Finland by the year 2020.

**Methods:**

The Delphi method was used, utilizing drug experts of the EU national network in Finland.

**Results:**

Marked growth was foreseen in drug use, especially in synthetic designer drugs and misuse of medicinal drugs. Significant increase was also expected in growing cannabis at home. However, the control of drug market was expected to shift more into the hands of organized crime. No consensus was reached on how drug prices will develop in the time period. Drug use is likely to remain punishable although the use and possession of cannabis may be treated less severely. It seems likely that health and social services resources will be directed towards medicinal treatment.

**Conclusions:**

Foresight can be utilized in preparing for the future; desirable developments can be fostered, and measures can be taken to curb probable but undesirable lines of development. Based on the results of this study, the experts’ view is that it is highly likely that the Finnish society will have to prepare for an increase in the demand for drug-related care, both in terms of content of the care and financing the services. Also, the forecasted increase in the role of legal prescription medicine used as intoxicants will call for efforts not only in changing prescription practices but in border and police control measures, as well. Parallel developments have been foreseen in the UK and Sweden, and it is likely that similar trends will actualize also in other western countries.

## Background

After a steady increase in the prevalence since the 1990s, lifetime prevalence of cannabis use in Finland is now 18.3% which is close to European average [1]. However, among 15-16-year-olds lifetime prevalence of cannabis use has been stable in 2000s, being now 11%. Lifetime prevalence of use of other illicit drugs in Finland is rather low: lifetime amphetamine use was 2.3%, and lifetime cocaine use 1.7% [1]. However, the prevalence of all drug use is notably higher among 25 to 34 year-olds [2]. Conspicuous to Finland, illicit buprenorphine is the drug of choice among opioid users [3]. Also other prescription opioids are widely abused, whereas heroin is almost non-existent in the Finnish drug market. Buprenorphine was reported as the main problem substance by 32% of the clients entering drug treatment in 2012, whereas only 1% reported heroin [4]. As in many other European countries, prevailing drug trends are increasing domestic cannabis cultivation as well as illicit import and use of so called designer drugs [1], [3]. The number of seizures of cannabis plants has increased tenfold since 2000: there were 3187 seizures in 2011 [1].

The Finnish drug policy has been characterized as a dual-track policy: both criminal control and health and social services have been advanced at the same time [5]. Finland has promoted harm reduction policies – e.g. opioid maintenance treatment and needle exchange – when simultaneously tightening criminal control measures and sanctions. The prevalence of HIV among IDUs has been low (<2%). Finland is not located on any major drug trafficking route, but drugs are smuggled into the country via all neighboring countries and by air and post [3].

For policy development purposes, it would be desirable to have some kind of outlook of the future. One of the leading tools in future science is the Delphi method: "Delphi may be characterized as a method for structuring a group communication process so that the process is effective in allowing a group of individuals, as a whole, to deal with a complex problem" [6]. Originating from the cold war era, the Delphi method was developed for defense technology forecasting [7]. Although Delphi and other expert panel methods for planning for the future are widely utilized in health and social sciences [8], [9], e.g. in alcohol policy development [10], their use within the field of drug abuse has been next to non-existent. Literature searches on psychoactive drug misuse foresight studies yielded only very few publications. Two of these publications were written by a single expert based on his insight [11], [12]. More recently, McBride et al. used Delphi to summarize experts’ views on reducing misuse of over-the-counter drugs [13]. The most notable drug policy foresight project has been the Drugs Futures 2025, a Technology Foresight Programme in the United Kingdom [14]. An editorial written by David Nutt [15] summarizes the main message as a future of growth in drug use: in addition to most of the current drugs of abuse still being around, new drugs with abuse potential will emerge by year 2025. A list of probable future developments in and around drug issues was presented together with possible options and their likely consequences for politicians to choose from [16]. Detailed predictions were made e.g. under the headings "Future medicines for mental health", "Future treatment for addiction", "The future of so-called 'recreational' drugs", "Future cognition enhancers", "Management of treatments for mental health", "Management of cognition enhancers for the healthy" and "Drug testing". The program has been criticized for not exploring the social and cultural contexts of drug use nor the drug users’ views, and presenting an unnecessarily gloomy future using “war against drugs” rhetoric [17]. A smaller scale foresight project was conducted in Sweden in 2010, bringing together the knowledge of around twenty experts [18]. The results were reported in one chapter in a book on the drug situation and included predictions such as general increase in use and harm resulting from use.

The Drugs 2020 study aimed at forecasting changes in the drug situation in Finland by the year 2020. The Delphi method was used with a drug expert network originally formed for European Union monitoring purposes. In addition to forecasting the probable future, the experts were requested to share their views on the desirability aspect of the predicted future. In contrast with survey studies, the experts also shared their argumentation behind the predictions. The discussion themes varied from drug use patterns to drug markets and from drug control to health and social care provision.

## Methods

Utilizing knowledge and skills of experts, the Delphi interaction process aims at producing a shared view on the issue at hand [19]. This shared view is likely to contain features on which most experts agree upon and elements where expert views differ. The "truth content" [20] is thought to increase as the number of participants increases; thus, small panels consisting of only a few experts are generally not favored. From a Leibnizian view, two essential qualities determine the ability of the communication process, such as the Delphi, to produce relevant information: the quality of the information held by the participants and the number of participants [20]. On the other hand, a Lockean stance on the truth content states that the method should be judged by its power to reduce complex issues to simpler statements and agreement on issues between different individuals [20]. Developments in the information technology have brought forward new ways of expert communication within the Delphi framework [20]. The traditional "wave" structure of the Delphi panel has recently somewhat dissolved and given way to continuous discussion that utilizes interactive web-based platforms [21]. This methodological extension is widely referred to as the "Real Time Delphi" [22]. In the current study, the project ideology came close to a Policy Delphi [23] as it did not aim at generating consensus but freely accepted discordance as a collective expert view. Drugs 2020 utilized a 'Real Time Delphi' -type [22] internet service called eDelfoi (http://www.edelphi.fi).

The European Monitoring Centre for Drugs and Drug Addiction (EMCDDA) coordinates a network of national focal points (NFPs) set up in the 27 EU Member States, Norway, the European Commission and in the candidate countries. The network is called Reitox, the European Information Network on Drugs and Drug Addiction. In Finland, the focal point was established in 1995 and the members of the national network of experts were used as informants in this Delphi study. The number of experts was 43 at the start of the study process, and these same experts comprise the national network in the European Monitoring Centre for Drugs and Drug Addiction. They have been assigned to this network to provide the European Union with comprehensive information on the drug situation in Finland. The experts came mainly from the public sector, but third sector actors were also involved from institutions like The National Institute for Health and Welfare, Statistics Finland, University of Helsinki, Ministry of Justice, Ministry of Social Affairs and Health, Ministry of Education and Culture, Customs service, National Bureau of Investigation (Police), City of Helsinki, Nordic Centre for Welfare and Social Issues, Finnish Medicines Agency, Finnish Society for Social and Health, the Helsinki Deaconess Institute and the A-Clinic Foundation. These institutions and organizations represent sectors involved in drug monitoring and policy planning in Finland. The experts are professionals in e.g. social sciences, medicine, chemistry, police, civil service, and law, and are in a position to estimate the development of Finnish drug situation.

The invitations to take part in the discussions were sent to the Finnish Reitox experts email list. It was emphasized that taking part was voluntary and absolutely anonymous. After registration to the eDelfoi website with an email address the participant could take part in the discussions. Only the eDelfoi site technical manager was able to access the email database. It was also emphasized that a temporary email address could be set up just for this purpose, in case the experts did not want to use their existing addresses. Thus, not even the researchers could know who answered what, let alone make it public. This was done in order to encourage honest personal views instead of so called official institutional views. The number of experts taking part in each of the three Delphi discussion rounds was 19, 18 and nine.

The three Delphi rounds were conducted during 2009–2011. The time for each discussion was limited to two weeks per round. The number of themes was limited to fifteen per round to encourage participation. Themes for the first Delphi round were developed within the research team. The second and third rounds included questions and topics formulated on ideas presented by the experts during the preceding rounds. Certain statements were re-issued on successive rounds, both in same wording and also in slightly developed form. This was done to clarify the issues in terms of evaluations of probability and justifications.

The majority of topics were dealt with by issuing a statement (e.g. 'Drug prices will decrease due to the supply exceeding demand.'), and requesting the respondent to choose between six alternatives: 'Highly likely', 'Likely', 'Not likely nor unlikely', 'Unlikely', 'Highly unlikely' and 'Cannot say'. In addition, probable prevalence of certain phenomena was inquired with questions like 'How common is the use of cocaine likely to be in the year 2020?'. The options ranged from 'Very rare (less than half of the current situation)' to 'At least three times as common as currently'. In conjunction with most topics, the desirability of the development was also inquired on a five-point scale ranging from 'Very desirable' to 'Not at all desirable', including the option 'Cannot say'. In four statements the desirability was presupposed as obvious, e.g. medicinal drugs causing more deaths, and thus the question of desirability was not asked. These cases are marked “na” in Table 1. In addition to inquiring about the likelihood and desirability of certain developments, the experts were also advised to justify their choices in their own words.

**Table 1:** The probability and desirability of changes until the year 2020

	**Probability**	**Desirability**
**Drug use and use patterns**		
The use of drugs is less common in 2020 than it is now.	–	na
The use of drugs has diminished due to population ageing.	–	++
The use of psychotropic medicine as intoxicants is more common than the use of illegal substances.	+	–
The use of new synthetic drugs is much more common in 2020 than it is now.	+	–
Use cultures and modes of use are more distinct and separated in 2020 and multi-drug use is less common.	–	|
**Drug markets**		
Drug prices decrease due to supply exceeding demand.	|	–
Drugs and psychotropic medicine parly replaces alcohol drinking.	–	– –
In 2020, drug import and wholesale is controlled by organized crime more than it is now.	+	na
**Drug control**		
Drug use and possession for own use is no longer illegal in 2020.	–	|
Use and possession of cannabis is no longer illegal in 2020.	|	|
In practice, use and possesion of cannabis is no longer punished in 2020.	|	|
Home growing cannabis for own use is no longer punishable in 2020.	|	|
The amount of designer drugs diminishes considerably due to new legislation speeding up their classification as illegal.	–	++
The Police no longer needs a court order to access telecommunications of suspected drug dealers.	|	|
A prison sentence due to drugs ordered by a court can be transformed into doing time in an intoxicant care institution.	+	+
**Health, care and the care system**		
A serious HIV epidemic has been experienced among injecting drug users by the year 2020.	–	na
The use of psychotropic medicine as intoxicants causes more deaths in 2020 than now.	+	na
Harm reduction has led to implementing use rooms for injecting drug users.	– –	|
A substitution therapy has been implemented widely for amphetamine addiction.	|	+
Resources in drug therapy have been focused in medicinal therapy.	|	–
Injecting drug users can be forced involuntarily to therapy in 2020.	–	–
Under-aged drug users can be forced involuntarily to therapy in 2020.	|	|
Drug therapy is offered almost entirely by the third sector.	|	–
Drug therapy is offered almost entirely by primary health care services in municipalities.	++	++
There are notably more combined mental health and drug addiction care units in municipalities in 2020.	+	+
Access to mental health care, including medication, has improved considerably in 2020, and this has diminished drug use.	–	++
**Attitudes toward drugs**		
Drug use approval has widened in sports, business and cultural circles.	+	– –
The society is markedly more negative towards drugs and the consequences are more severe than now.	|	|

Probability: "--" no-one agreed, "-" more than two thirds disagreed, " + " more than 2/3 agreed, "++" no-one disagreed.

Desirability: "--" no-one held desirable, "-" more than two thirds held undesirable, " + " more than 2/3 held desirable, "++" no-one held undesirable.

"|" no agreement on whether the issue is probable (desirable) or not probable (undesirable).

"na" not applicable, was not asked.

In contrast with survey questionnaires, the web-based response platform was open for the experts to view others’ responses and adjust their own during the two week window for each discussion round. The Delphi method aims at promoting deliberation and making informed predictions on the future, and thus encourages information sharing. The experts were able to view the preliminary results, both quantitative and qualitative. They were also able to amend or change their own views in case other justifications made sense to them.

## Results

### Drug use and use patterns

All in all, the use of drugs was predicted to be more widespread in Finland by the year 2020 compared to current situation (Table 1). Use of psychotropic medicine as intoxicants, as well as use of new synthetic drugs was seen as areas of particular growth. In their open-ended justifications the respondents explicated that most drug users reason that drugs manufactured by the pharmaceutical industry are both safer and easier to access. In the case of new synthetic drugs, both the increased access to process chemicals through internet and the instructions available online were seen as particular driving forces behind the predicted increase. Both of these phenomena were considered undesirable, although a number of respondents noted that medicinal drugs may cause less harm than illegal substances. However, other respondents brought forward that potential disappearance of the differentiation between medicinal drugs and drugs of abuse may encourage the use of medicine to non-medical purposes. The experts unanimously dismissed the idea that drug use would become less common due to population ageing - this was considered to be wishful thinking.

On the first Delphi round, the experts were asked how common they thought the use of cocaine would be in 2020 compared to the current prevalence (=100%). Half of the respondents predicted the use of cocaine to be somewhat more common than now. When the same question was presented to them on the second round, two thirds thought the use will have increased. Half of the experts predicted slight increase (less than 50% increase) and no one foresaw greater than two-fold increase. According to the respondents’ justifications, the potential for increase was seen to be rather small due to the strong culture of amphetamine use in Finland. However, the respondents admitted that cocaine is much more popular in most other European countries, and this causes obvious pressure for increase also in Finland. It was also noted that the growth seen in designer drugs may well override the potential for cocaine use increase.

On both the first and the second Delphi round the experts were asked to estimate "How common growing cannabis at home will be in 2020 compared to now?". The majority expected increase; the first time the mode was "50% more than now" whereas the second time the mode was "100% more than now". Only few saw the phenomenon as something which may be popular now but will go away soon. The respondents’ view was that home growing is seen to have so many benefits for the users compared with buying cannabis from criminal market that it would be very difficult to counteract this with increased control. The experts also noted that the risk of being caught is not high and not likely to grow.

### Drug market

The control of drug market in Finland was expected to shift more into the hands of organized crime (Table 1). The respondents expressed that this is partly due to the anticipated growth of the market which will attract larger scale players to the scene. Furthermore, they pointed out that existing organizations are already well networked with the international drug trade. The minority of the respondents, who thought this kind of development not probable, justified their thinking by hope that drug control will be directed more towards criminal organizations as opposed to individual users.

No consensus was reached on how drug prices will develop in the time period. Those expecting a price decrease thought that home-grown cannabis and cheap synthetic drugs are likely to increase the supply of drugs. Those with the opposing view saw that organized crime will act like a monopoly and keep prices steady or even increasing.

### Drug control

The experts were markedly divided in their views on the future of drug control issues in Finland (Table 1). More than half of the drug control statements were considered neither probable nor improbable. Furthermore, the experts did not agree on whether the suggested developments were desirable or undesirable, except on two topics. Speeding up the classification of new synthetic substances as illegal drugs was strongly desired to help curb the increase in usage; however, the majority of the experts did not think this legislative action could do the job because of the infinite potential to alter these substances. Most of the experts were in favor of making it possible to transform a drug-related prison sentence into therapy period in an intoxicant care institution - and the majority thought this is likely to happen by the year 2020.

### Health, care and the care system

Among the experts, strong consensus existed on what the main institution bearing the biggest load in drug user health care would be: most likely the local municipal primary care services (Table 1). Furthermore, experts were unanimous that this is a desirable way to go. Of the alternatives, specialized health care was seen as unnecessarily expensive. The respondents’ view was that the number of patients with drug-related health problems has been on the increase and is likely to further increase, and thus the expertise on drugs in the primary health services will have to grow. Currently, third sector bodies such as non-profit organizations and foundations provide a notable part of these services, but an expansion of their role was considered neither probable nor desirable. The experts also envisioned that the municipal care services are likely to develop into integrated mental health units incorporating drug services.

Regarding intravenous drug users, injection rooms were considered a very unlikely development in Finland, as was the idea of forced involuntary therapy. The experts' views were strongly divided in predicting the following developments: replacement therapy for amphetamine users, medicalization of care, and involuntary therapy for under-age users. On one hand, amphetamine replacement therapy was seen as a desirable opportunity but, on the other hand, concentrating on medicinal therapy was thought as an undesirable development: this view was based on experiences with opioid substitution, more precisely the so-called substitution therapy dependence. Although not necessarily likely, the possibility to force therapy on the under-age users would be welcomed by the experts.

The majority of experts estimated that the amount of people in opioid replacement therapy would grow in the coming years; the estimate of a 50% increase seemed most likely. It was noted that even if opioid use did not become more common in Finland, more services would be needed since not nearly all care demands are met currently. Furthermore, therapy is long-lasting, and few actually ever stop using opioids.

### Attitudes towards drugs

The experts did not see any strong trend in public attitudes towards drugs in the future (Table 1). However, a slight shift to more permissive attitudes was envisioned. The respondents’ main argument was that in most areas of life competition is getting tougher and, therefore, new means for success are sought and at least quietly approved of. The opponents to this view referred to the development seen in smoking: smokers are increasingly seen as “pathetic losers”, as one expert phrased it. Regarding the desirability of attitude change, the experts were unanimous in condemning any change towards wider approval. More widespread approval of drugs was seen to promote increase in drug use. The experts did not favor hardening the consequences, especially criminal sentences.

## Discussion

The Drugs 2020 study aimed at forecasting changes in the drug situation in Finland by the year 2020. The Delphi method was used, and ideology of the study came close to a Policy Delphi as it did not aim at forced consensus but freely accepted discordance as a collective expert view. The experts agreed on the view that drug use is likely to become more common in Finland by 2020. Marked growth was foreseen especially in synthetic designer drugs and misuse of medicinal drugs. Significant increase was also expected in growing cannabis at home. However, the control of drug market was expected to shift more into the hands of organized crime by 2020. Regarding criminal sanctions, the use and possession of cannabis may be treated less severely by 2020 – this being a highly debated issue in Finland, as well as in most other western countries.

When comparing views of other countries, the Finnish experts seemed rather conservative in their predictions, as were their Swedish colleagues [18]. In fact, the Swedish experts foresaw future developments, such as general increase in use as well as marked increase in intoxicant use of prescription medicine and synthetic drugs, in much the same way as their Finnish counterparts. Drug legalization processes are not likely to advance in Sweden, although the experts pointed out that there will be increased pressure towards this development from neighboring countries [18] – Finnish experts predicted exactly the same. Another mutually shared view was on the likelihood of medicinal care growing at the expense of psycho-social care: in light of current economic situation, cutting public spending is likely to speed up this line of development. All in all, the future predicted for Sweden [18] seemed strikingly similar to the one foreseen for Finland by the Finnish experts.

In the UK, on the other hand, experts were notably more innovative in their predictions of the future, possibly partly due to the longer prediction time-frame. One other likely explanation lies in the differences in current drug situations: whereas Finland and Sweden can be considered countries with limited and controlled drug use, the situation in the UK is further advanced and dynamic. Furthermore, the UK foresight study seemed to emphasize out-of-the-box thinking, and encouraged the experts to think in terms of possibilities for innovation alongside threats [14]. Thus, the experts came out with predictions such as vaccines for addictions, new legal psychoactive substances for recreational use, and cognition enhancers for use in the working life [24]. However, following the lines of the Finnish foresight project, experts in the UK thought it likely that drug-related deaths will become more common and drug crime will increase.

Foresight settings, research questions and methodologies have often been evaluated as poorly performed surveys or inadequate prospective policy studies. In contrast with other methods, the main logic behind a Delphi expert panel is facilitating structured expert communication on an issue that evades study when traditional methods are used. The Delphi method has been considered useful when means of establishing and articulating the views of experts is needed [25], [26]. The methodological considerations in a Delphi study are different from both survey studies and interview studies; for example, the method calls for a heterogeneous set of experts to ensure that conflicting views can be identified. As a reaction to criticism on one-sided thinking in the UK futures project [17], for our study we invited experts representing a wide variety of institutions and professions. On the first and second discussion rounds, roughly half of the eligible experts participated in the process. The third round failed to inspire the experts to participate, possibly since the most important questions had already been covered. We have no way of knowing who the respondents were, but we can still be sure that, even with the low response on round three, the diversity in expertise was retained at least to some degree as none of the institutions were represented by a numerous amount of experts. Although, due to anonymity we cannot explicitly study the qualities of the respondents versus those who did not participate, we did receive voluntary information from some eligible experts on their non-participation. A common explanation for not taking part was a feeling of inadequacy. Many felt they were experts in such a narrow field that they did not feel competent to take part in a discussion on wider issues; an example would be a chemist analyzing cannabinoid content of confiscated specimen. It is unlikely that any one person could be an expert on all issues and aspects of the future of drugs, and we judged that the EU national network of experts was and is the best existing collection of experts available.

Although this Delphi study on the probable future related to drugs in Finland was based on an established network of experts, brought together for European Union drug situation monitoring purposes, other experts like many academically oriented researchers were not included in the process. Clinicians involved in the treatment of drug users were not included, nor were drug users themselves. Thus, it must be remembered that the view on a probable future depicted in this study is likely to be an institutional view rather than a view of an individual. It is also worth noting that seeing the future as an extension of current trends is the easiest mode of thinking [27]. Many of the predictions made in the current study for Finland, as well as in the Swedish study [18], can be classified as extrapolations of past trends.

## Conclusions

Foresight can be utilized in preparing for the future: desirable developments can be fostered and measures can be taken to curb probable but undesirable lines of development. Based on the results of our study, the experts view is that it is highly likely that the Finnish society will have to prepare for an increase in demand for drug-related health care, both in terms of care content and financing the services. As it is likely that the amount of drug users and demand for health care will increase, the experts saw that the emphasis in care will have to shift away from specialized services nearer to the citizens, to primary care. Also, the likely increase in the role of legal prescription pharmaceuticals used as intoxicants will call for efforts not only in changing prescription practices but in border and police control measures, as well. The most topical development need relates to a process already started in Finland involving a centralized electronic database for prescriptions. This database can be utilized to detect and limit the sales of drugs with potential for misuse. Even if the national drug policy is to remain the same, i.e. based on both criminal sanctions and on health and social services [4], the results of our study call for a more open discussion on policy objectives and practical measures to control both the use of drugs and harm caused by them. Parallel developments were foreseen in the foresight studies conducted in the UK [14] and Sweden [18], and it is likely that similar trends will actualize also in other western countries.

## Competing interests

The authors declare that they have no competing interests.

## Authors’ contributions

TL conceived of the study, supervised the design and data collection, conducted the numerical analyses and wrote the first version of the manuscript. AK participated in the study design, formation of the statements and interpretation of the results. SR acted as the coordinator of the expert panel and participated in the formation of the Delphi statements. EK participated in the formation of the final Delphi round statements and interpreting the results. All authors read and commented on the manuscript, and approved the final manuscript.
